# CircAGAP1 promotes tumor progression by sponging miR-15-5p in clear cell renal cell carcinoma

**DOI:** 10.1186/s13046-021-01864-3

**Published:** 2021-02-22

**Authors:** Qi Lv, Gangmin Wang, Yinan Zhang, Aijun Shen, Junjun Tang, Yi Sun, Chunhui Ma, Peijun Wang

**Affiliations:** 1 Department of Medical Imaging, Tongji Hospital, Tongji University School of Medicine, Xincun Road No. 389, Shanghai, China; 2 Department of Urology, Huashan Hospital, Fudan University, Urumuqi Road No. 12, Shanghai, 200040 China; 3 Department of Urology, Shandong Provincial Hospital affiliated with Shandong University, Jingwuweiqi Road No. 324, Jinan, 250021 Shandong China; 4 Department of Orthopedics, Shanghai General Hospital of Shanghai Jiaotong University, Wujin Road No. 85, Shanghai, 200080 China; 5 Department of Urology, No.971 Hospital of the PLA Navy, Qingdao, 266071 Shandong China

**Keywords:** Clear cell renal carcinoma, circRNA, miR-15, E2F3, ceRNA

## Abstract

**Background:**

Accumulating evidence has revealed that circular RNAs (circRNAs), as novel noncoding RNAs, play critical roles in carcinogenesis and tumor progression. However, the functions and molecular mechanisms of circRNAs in clear cell renal cell carcinoma (ccRCC) are largely unknown.

**Methods:**

The expression and functions of circAGAP1 were identified in clinical samples, ccRCC cells and in vivo animal models. The molecular mechanism of circAGAP1 was investigated by fluorescence in situ hybridization, RNA immunoprecipitation and luciferase assays.

**Results:**

circAGAP1 (circ0058792) expression was significantly upregulated in ccRCC tissues compared to adjacent nontumor tissues. Moreover, the expression of circAGAP1 was closely related to the tumor size, nuclear grade and clinical stage of ccRCC in patients. Mechanistic studies demonstrated that cytoplasmic circAGAP1 targeted miR-15-5p in an RNA-induced silencing complex. Additionally, miR-15-5p expression was downregulated in ccRCC. Luciferase reporter assays showed that E2F transcription factor 3 (E2F3) was a target of miR-15-5p, and upregulated E2F3 expression was positively correlated with circAGAP1 in ccRCC. Furthermore, the tumor-promoting functions of circAGAP1 could be alleviated by miR-15-5p mimics in vitro and in vivo.

**Conclusion:**

Our results clarify that circAGAP1 exerts its oncogenic functions as a competitive endogenous RNA (ceRNA) by sponging miR-15-5p, which promotes E2F3 expression. Targeting circAGAP1 might be a new attractive therapeutic strategy in ccRCC.

**Supplementary Information:**

The online version contains supplementary material available at 10.1186/s13046-021-01864-3.

## Background

The incidence of renal cell carcinoma has increased annually (approximately 7% per year) over the past years [1]. Clear cell renal cell carcinoma (ccRCC) has the highest mortality among all RCC subtypes. Although there has been progress in the clinical treatment for RCC, the prognosis of RCC at an advanced stage is still poor, and the expected efficacy of targeted therapy is unsatisfactory due to drug resistance and severe adverse reactions [2, 3]. Thus, it is crucial to clarify the molecular mechanisms and to screen novel biomarkers for ccRCC.

Circular RNAs (circRNAs), a recently discovered type of endogenous noncoding RNAs, are covalently closed loops with 5′-caps and 3′-poly(A) tails joined together [4]. Recently, the aberrant expression of circRNAs has been shown to contribute to the development and progression of malignant tumors, such as glioblastoma [5, 6] and hepatocellular carcinoma [7, 8]. Moreover, multiple functions of circRNAs have been recognized, especially as competitive endogenous RNAs (ceRNAs) to regulate genes by sponging miRNAs [8]. miR-15 has been shown to control diverse molecular processes [9–11]. In chronic lymphocytic leukemia cells, miR-15 has been reported to suppress proliferation by targeting Bcl2 [12], whereas in neuroblastoma and pancreatic ductal adenocarcinoma, miR-15 regulates growth by modulating the expression of multiple targets [13, 14]. However, there is no report on the role of miR-15 in ccRCC.

E2F transcription factor 3 (E2F3) promotes carcinogenesis [15]. E2F3 expression correlates with prognosis in a variety of cancers, highlighting the importance of E2F3 in determining the clinical cancer phenotype [16]. Several tumor-related miRNAs have been found to target E2F3, including miR-128 [17], miR-377 [18], and miR-34a [19, 20]. Although E2F3 mediates cancer cell proliferation, angiogenesis, antiapoptotic activity, invasion and metastasis, the functions and underlying mechanisms of E2F3 in ccRCC are still unknown.

Thus, based on a previous circRNA microarray and quantitative real-time polymerase chain reaction (qRT-PCR) in ccRCC tissues, we found for the first time that the expression of circAGAP1 (circ0058792), which is located in exons 2 to 6 of the AGAP1 gene, is upregulated in ccRCC and participates in tumorigenesis by regulating proliferation, migration, invasion and apoptosis. We also found that circAGAP1 could sponge miR-15a-5p to upregulate E2F3 expression and subsequently promote the progression of ccRCC. In summary, circAGAP1 is a novel oncogene that might be a potential therapeutic target in ccRCC.

## Methods

### Patients and tissue samples

Thirty-four pairs of fresh-frozen ccRCC samples and matched nontumor tissues were obtained from the Urology Department at Tongji Hospital (Shanghai, China) and Shandong Provincial Hospital (Jinan, China) from June 2015 to July 2016. The inclusion criteria for the study subjects were as follows: (1) pathologically confirmed ccRCC; (2) no prior adjuvant treatment before radical resection; and (3) complete follow-up data. The exclusion criterion was a diagnosis of other types of renal carcinoma, such as chromophobe cell carcinomas and renal papillary carcinoma. All patients were subject to clinical, imaging, and pathological diagnosis (Supplementary Table 1). All the samples were immediately submerged in liquid nitrogen for storage. Clinical information was collected at Tongji Hospital. The characteristics of the patients are shown in Supplementary Table 1. The study was reviewed and approved by The Ethics Boards of Tongji Hospital and carried out in accordance with institutional guidelines, and informed consent was obtained from the patients undergoing surgery.

### Cell lines and cell culture

Human ccRCC cells (ACHN and A498) were obtained from GeneChem (Shanghai, China), and CAKI-1, OS-RC-2 and normal HK-2 cells were purchased from iCell Bioscience Inc. (Shanghai, China). HK-2 cells were maintained in iCell-0019 medium, whereas ACHN, A498, CAKI-1 and OS-RC-2 cells were maintained in Dulbecco’s modified Eagle medium (DMEM) supplemented with 10% fetal bovine serum (FBS) and penicillin/streptomycin; all cells were cultured at 37 °C in a humidified incubator with 5% CO_2_.

### Cell transfection

CircAGAP1 (hsa_circ_0058792) cDNA was cloned into pcDNA3.1 by Hanbio (Guangzhou, China). miR-15a-5p mimic and corresponding control (NC) were purchased from GenePharma (Shanghai, China). Transfection of plasmids, small interfering RNAs (siRNAs), miR-15a mimics or their corresponding NCs into cells was performed using Lipofectamine 2000 (Invitrogen). circAGAP1 siRNA (5′-GACGATGCCTTCGTGAACA-3′) and control siRNA (5′-TTCTCCGAACGTGTCACGT-3′) were produced by GenePharma (Shanghai, China).

### Circular structure confirmation

The circular structure of circAGAP1 was confirmed by RNase R treatment and Sanger sequencing. For RNase R treatment, 5 μg of RNA was digested with 10 U RNase R (GENESEED company, Guangzhou, China) at 37 °C for 30 min. The levels of circAGAP1 and linear AGAP1 mRNA were examined using quantitative real-time PCR (qRT-PCR).

### Quantitative reverse transcription polymerase chain reaction

RNA from ACHN or A498 cells was extracted via TRIzol (Invitrogen) and reverse transcribed with primers. The specific primers used were as follows: E2F3-F, 5′-AAAGCCCCTCCAGAAACAAGA-3′; E2F3-R, 5′-CCTTGGGTACTTGCCAAATGT-3′; GAPDH-F, 5′-GTCTCCTCTGACTTCAACAGCG-3′; GAPDH-R, 5′-ACCACCCTGTTGCTGTAGCCAA-3′; circAGAP1-F, 5′-GTCTTCCAGGACGATGCCTT-3′; circAGAP1-R, 5′-GCTGGCCAAGTTACCCACAA-3′; U6-F, 5′-TGGAACGCTTCACGAATTTGCG-3′; U6-R, 5′-GGAACGATACAGAGAAGATTAGC-3′; hsa-miR-15a-5p-F, 5′-ACTCCAGGGCTACAACTGGTCGTGGAGTCGGCAATTCAGTTGAGCACAAACC-3′; and hsa-miR-15a-5p-R, 5′-ACACTCCAGGGCGGCAACTGGTGTCGCAAT-3′.

### Cell proliferation

For the Cell Counting Kit-8 (CCK-8) assay, the cells were plated at 3000 cells/well in 96-well plates. Then, 10 μL of CCK-8 (Dojindo, Japan) was added to each well, and the optical density (OD) at 450 nm was collected every day until day 5. The 5-ethynyl-20-deoxyuridine (EdU) incorporation experiment was completed according to the manufacturer’s recommendations (Ruibo, China).

### Migration and invasion experiments

Transwell inserts (Corning, NY, USA) with and without Matrigel were used to assess the invasion and migration of cells, respectively. In brief, a suspension comprising 5 × 10 ^ 5 cells/mL was prepared in serum-free medium and 200 μL were added to the upper insert of the Transwell. Approximately 700 μL of complete medium was plated in the lower chamber of the Transwell and incubated for 24 h. The membrane in the upper insert of the chamber was rubbed gently with a cotton swab to remove any nonmigrating cells before it was fixed in 4% methanol for 20 min, stained with 400 μL of 1% crystal violet staining solution and incubated for 15 min at room temperature to stain cells that passed to the lower chamber. The cells were photographed, and the cell numbers were calculated in at least three separate fields of view.

### Apoptosis assays

ACHN and A498 cells (5 × 10^5^ per well) were transfected as indicated in 6-well plates. Cells were collected and stained with Annexin V-FITC and propidium iodide (PI) (BD Biosciences, San Jose, CA, USA). Briefly, cells were suspended in 200 μL of binding buffer mixed with 5 μL of Annexin V and 5 μL of PI and cultured for 15 min at room temperature. The procedures were carried out according to the kit instructions. A flow cytometer (BD Pharmingen, San Diego, CA, USA) was used to measure the numbers of apoptotic ACHN and A498 cells.

### Luciferase assay

PmirGLO dual-luciferase reporter plasmids (Promega) were used for luciferase reporter analysis. Wild-type circAGAP1 (circAGAP1-WT) and mutant circAGAP1 (circAGAP1-MUT), which had mutations at the potential miR-15a-5p binding sites, were amplified by PCR and cloned into the pmirGlo dual-luciferase vector with restriction sites of SacI and XhoI to construct a luciferase reporter vector (pmirGLO-circ_0015756-WT and pmirGLO-circ_0015756-mut, respectively; GeneChem Co., Shanghai, China). Similarly, WT E2F3 (pmirGLO-E2F3-WT) or E2F3 with mutations at the potential miR-15a-5p binding sites (pmirGLO-E2F3-MUT) were also designed by Shanghai GeneChem Co.

The reporter plasmids were cotransfected with miR-15a-5p mimic or control mimic (mimic NC) with Lipofectamine 3000 (Thermo Fisher Scientific) and cultured further for 48 h. Luciferase activity was analyzed by the dual-luciferase reporter assay (Promega). Experiments were performed in triplicate.

### Fluorescence in situ hybridization (FISH)

FISH was performed to investigate the distribution of circAGAP1 and miR-15a-5p in ACHN and A498 cells. In brief, Cy3-labeled probes targeting miR-15a-5p (hsa-miR-15a-5p-CY3: CAAACCATTATGTGCTGCTA) and FITC-labeled probes targeting circAGAP1 (hsa_circ_0058792-FITC: TTCACGAAGGCATCGTCCTGGAA) were designed. FISH analysis was performed using a Fluorescent In Situ Hybridization Kit (GENESEED, Guangzhou, China) according to the manufacturer’s instructions. Nuclei were counterstained with DAPI (Beyotime, China). Images were obtained under laser scanning confocal microscopy (Leica, Germany).

### RNA pull-down

An in vitro biotin-coupled probe RNA pull-down was performed by GeneCreate Biotech (Wuhan, China). The sequences of the biotin-labeled circRNA and control probe were UGUUCACGAAGGCAUCGUCCUGGAAGACCC-biotin and biotin-AAACAGTACTGGTGTGTAGTACGAGCTGAAGCT (control oligo probe). ACHN cells were lysed and incubated with magnetic beads coated with the circAGAP1-targeting biotin-coupled probe. Then, the RNA was purified with TRIzol (Invitrogen). circAGAP1 and miR-15a-5p enrichment was determined by qPCR.

### Western blotting

Proteins were extracted from cells with RIPA buffer (Beyotime, China) and quantified by a Coomassie Blue assay (Pierce). Proteins (50 μg) were resolved by SDS-polyacrylamide gel electrophoresis (10%) and transferred onto Hybond ECL membranes (Amersham). The membranes were blocked in 5% skim milk/TBS-T at room temperature (RT) for 45 min and then incubated overnight at 4 °C with antibodies against E2F3 (cat. no. DF12390, AFFINITY), N-cadherin (cat. no. 13116 T, Cell Signaling Technology), proliferating cell nuclear antigen (PCNA) (cat. no. AB0051, Abway), Bcl2 (cat. no. 2870 s, Cell Signaling Technology) and β-actin (cat. no. ab8226, Abcam) at a 1:1000 dilution. Then, the membranes were incubated with horseradish peroxidase (HRP)-conjugated secondary antibodies (anti-rabbit or anti-mouse antibody, Sigma) for 1 h at RT. Protein bands were visualized by Western blot detection reagents (Millipore).

### Xenograft tumor model

The animal study was approved by the Institutional Animal Care and Use Committee of Tongji Hospital, Tongji University (2019tjdx210). ACHN or A498 cells (1 × 10^7^ per mouse) were subcutaneously injected into athymic nude mice (4 weeks old). After 4 weeks, mice with established tumor were assigned into three groups and intratumorally injected with siRNA NC (5 nmol), circAGAP1 siRNA alone (5 nmol) or circAGAP1 siRNA (5 nmol) combined with miRNA-15a-5p inhibitors (10 nmol). Tumor size and final tumor weight were recorded.

### Immunohistochemistry (IHC)

Deparaffinized and rehydrated tissue sections were heated in citrate buffer (pH 9.0) at 98 °C for 20 min and cooled to RT for antigen retrieval. Then, sections were cultured with primary antibodies and respective secondary antibodies (listed in the Western blotting section), further stained with streptavidin–biotin–peroxidase reagents and counterstained with hematoxylin.

### Statistical analysis

SPSS 17.0 (SPSS Inc., Chicago, USA) was used. The data are shown as the means ± standard error of the mean (SEM) and were normally distributed as examined by the Shapiro-Wilk test. An independent-samples t test was also applied to define correlations between circAGAP1 expression and the clinicopathological parameters of ccRCC patients. The graphs were constructed using GraphPad Prism 5.0 (GraphPad Software, La Jolla, CA). A paired-samples *t* test was used to compare circAGAP1 expression in cancerous and noncancerous tissues. *P* < 0.05 was statistically significant.

## Results

### circAGAP1 (circ0058792) is highly expressed in ccRCC

**Fig. 1:**
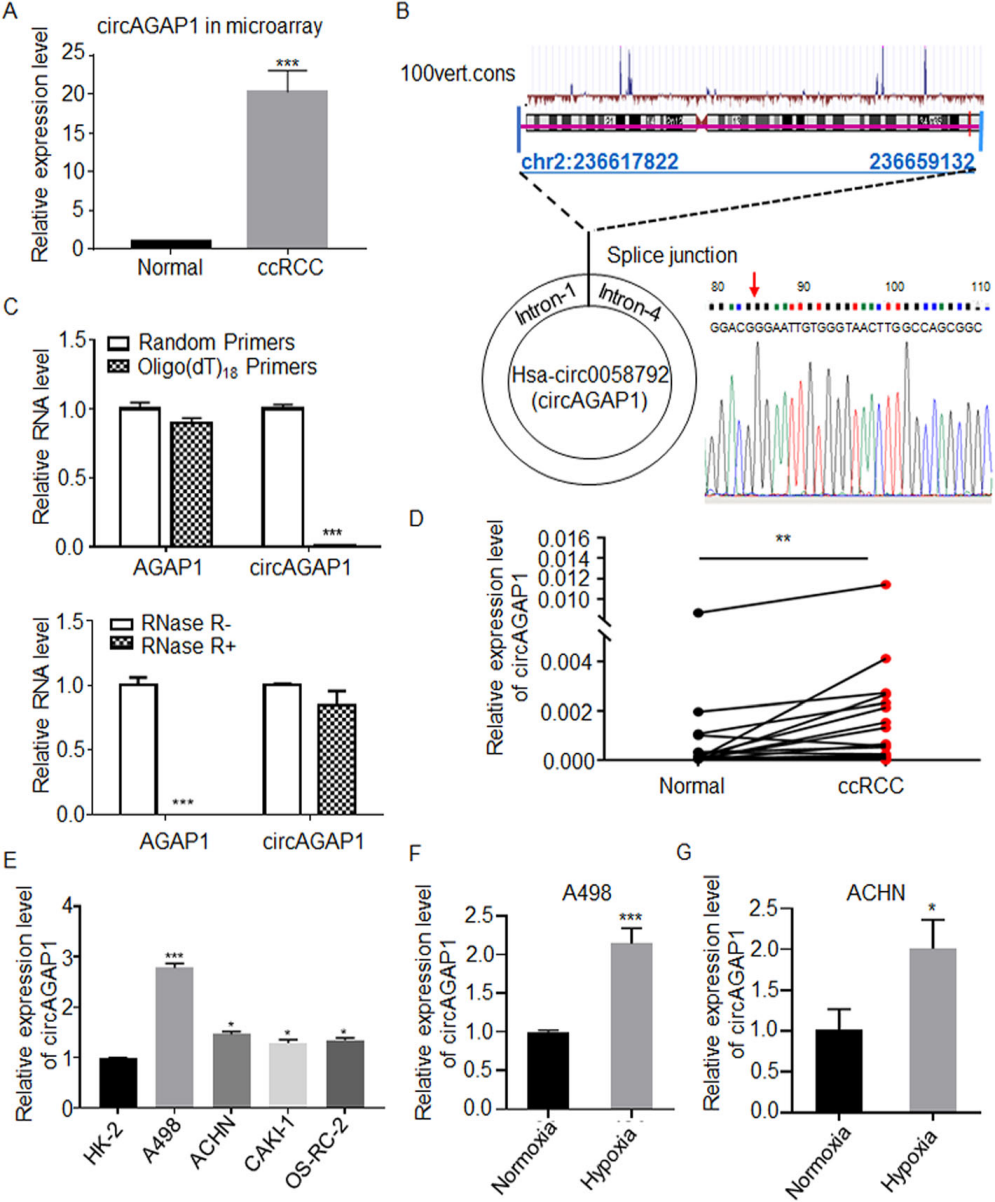
Expression and characterization of circAGAP1 in ccRCC. **a** The expression of circAGAP1 in ccRCC was examined by microarray. **b** The exonic information of circAGAP1 (circBase ID: hsa_circ_0058792) is demonstrated. Hsa_circ_0058792 is generated by circularization of exons 2–6 of the AGAP1 gene. The back-splicing junction was verified via Sanger sequencing. **c** Random hexamer or oligo (dT)18 primers were used for the reverse transcription assay. The RNA levels of circAGAP1, linear AGAP1 and GAPDH were detected by RT-qPCR in samples with RNase R (RNase R+) or without RNAse R (RNase R-) treatment. Data are presented as the means ± SEM from three independent experiments. **d** Expression levels of circAGAP1 in paired ccRCC and adjacent noncancerous tissues (*n* = 34). **e** qRT-PCR analysis of circAGAP1 expression in human ccRCC cell lines (A498, ACHN, CAKI-1 and OS-RC-2) and HK2 cells. **f**, **g** qRT-PCR analysis of circAGAP1 expression in human ccRCC cells (A498, ACHN) subjected to either normoxia or hypoxia for 12 h. Data are presented as the means ± SEM from three independent experiments and are expressed as the relative fold-change over normoxic controls. *p < 0.05, **p < 0.01, ***p < 0.001

We employed paired ccRCC and adjacent nontumor tissues from 4 patients for circRNA microarray analysis. We found that many circRNAs were abnormally expressed in ccRCC, among which circ0058792 was one of the most upregulated circRNAs (fold change =21.42, *p* value< 0.001, Fig. 1a**)**. A search of circBase (http://www.circbase.org/) showed that circ0058792 is located at chr2:236617822–236,659,132 with a sequence length of 510 bp and is generated from exons 2–6 of AGAP1 **(**Fig. 1b). circ0058792 was successfully amplified by random primers but not by oligo (dT) primers **(**Fig. 1c). Moreover, RNase R exonuclease treatment revealed that circ0058792 was much more stable than the linear transcripts of AGAP1 mRNA, which were degraded by RNase R exonuclease (Fig. 1c). We further verified the back-splicing junction via Sanger sequencing **(**Fig. 1b**)**. Thus, these are the first data indicating the existence of circAGAP1 (circ0058792), which is highly expressed in ccRCC tissues.

To determine the clinical importance of circAGAP1, the expression of circAGAP1 in ccRCC tissues was further analyzed with paired ccRCC tissues and adjacent nontumor tissues (*n* = 34). The results revealed that circAGAP1 expression was significantly increased in ccRCC tissues compared to nontumor control tissues, which is consistent with the microarray data (Fig. 1d). Moreover, circAGAP1 expression was upregulated in an array of ccRCC cells compared to HK-2 cells (Fig. 1e). Considering that tumor cells often lack sufficient oxygen under physiological conditions, we showed that ccRCC exhibited increased circAGAP1 expression under hypoxic conditions (Fig. 1f and g), which may indicate that circAGAP1 plays an important role in ccRCC. As shown in Table S2, elevated circAGAP1 levels were significantly associated with tumor size, nuclear grade and clinical stage. Collectively, these results suggest that upregulated circAGAP1 is significantly associated with worse clinicopathological factors in ccRCC.

### circAGAP1 enhances the viability, invasion and survival of ccRCC cells

**Fig. 2:**
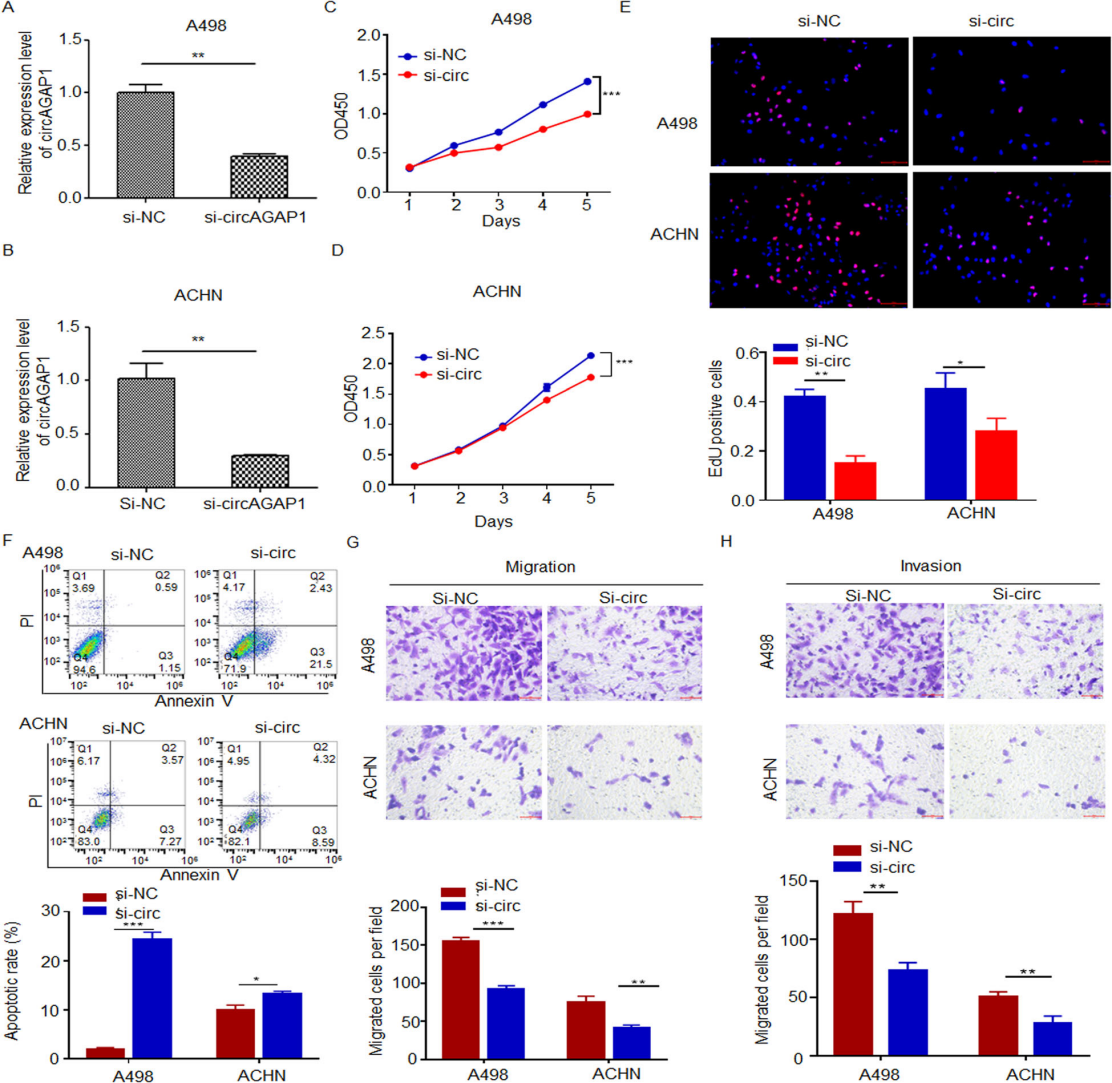
Suppression of circAGAP1 expression reduced the proliferation, migration and invasion of ccRCC cells. **a, b** qRT-PCR analysis of circAGAP1 in A498 and ACHN cells transfected with si-circAGAP1 or si-NC. Data are presented as the means ± SEM from three independent experiments. **c, d** The viability of A498 and ACHN cells transfected with si-circAGAP1 or si-NC was determined by the CCK-8 assay. **e** EdU analysis and quantification of EdU-positive A498 and ACHN cells transfected with si-circAGAP1 or si-NC. **f** Apoptotic rates of A498 and ACHN cells transfected with si-circAGAP1 or si-NC as detected by flow cytometry. **g, h** Transwell assays of A498 and ACHN cells transfected with si-circAGAP1 or si-NC. The experiments were repeated with at least three biological replicates. *p < 0.05, **p < 0.01, ***p < 0.001

To clarify the functions of circAGAP1 in ccRCC, we analyzed circAGAP1 expression in ccRCC cell lines. Among the cell lines tested, A498 and ACHN cells showed high levels of circ0058792 expression (Fig. 1e). Thus, siRNAs against circAGAP1 were constructed. The expression of circAGAP1 was significantly suppressed in A498 and ACHN cells transfected with circAGAP1 siRNA, whereas AGAP1 mRNA levels displayed no significant alterations (*P* < 0.01, Fig. 2a and b, Supplementary Fig. 1a).

The CCK-8 assay revealed that the viability of ACHN and A498 cells was reduced after circAGAP1 knockdown compared to that of the control cells (*P* < 0.001, Fig. 2c and d). Consistently, the EdU experiments showed that the numbers of EdU-positive cells were reduced in the circAGAP1-knockdown groups (*P* < 0.001, Fig. 2e). Flow cytometry analysis was performed to evaluate whether circAGAP1 affects ccRCC cell viability by altering cell cycle progression and apoptosis. The results showed that circAGAP1 suppression significantly increased the percentage of apoptotic cells (*P* < 0.001, Fig. 2f). Moreover, the migration and invasion abilities were reduced in cells with circAGAP1 knockdown (*P* < 0.001, Fig. 2g and h).

**Fig. 3:**
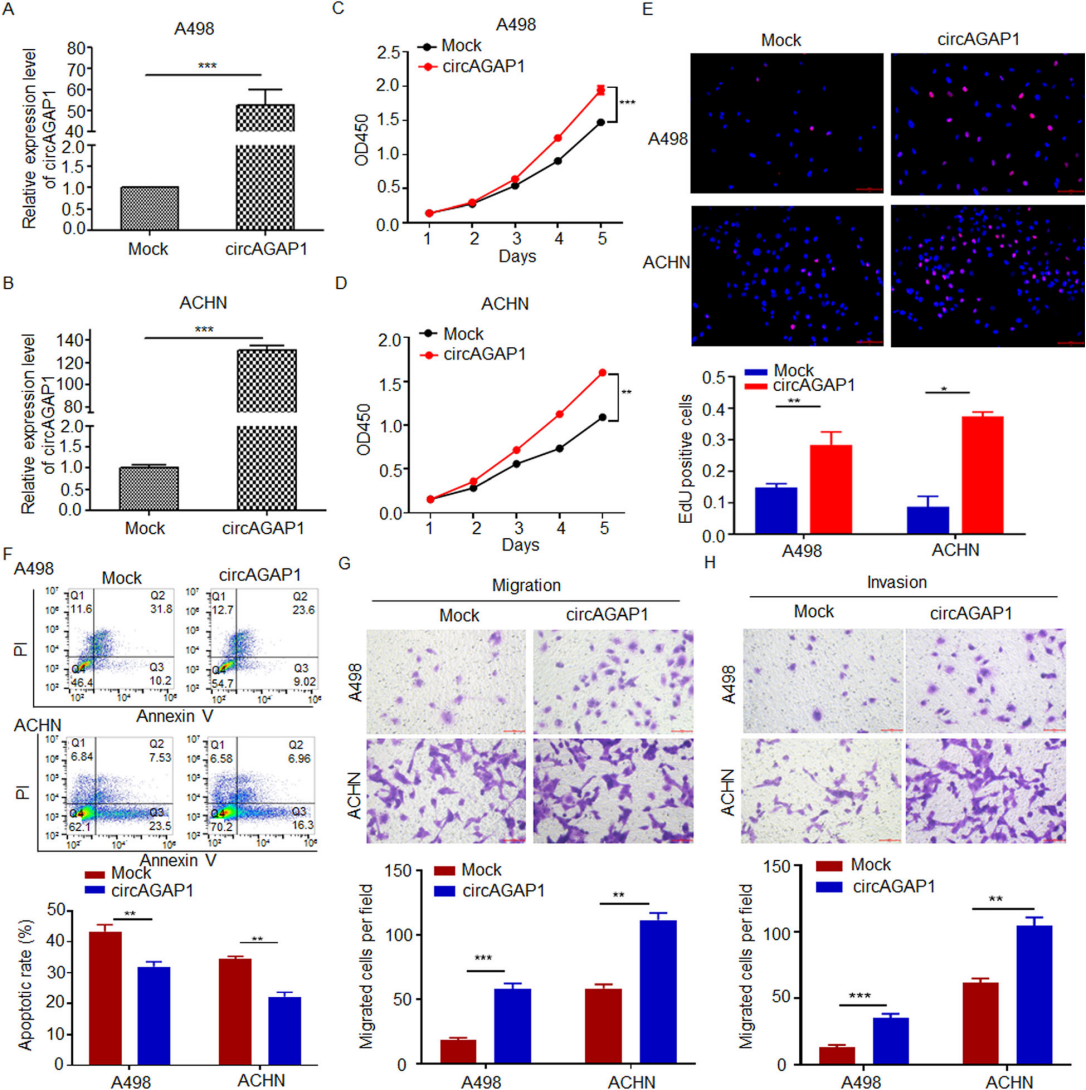
Overexpression of circAGAP1 promoted the proliferation, migration and invasion abilities of ccRCC cells. **a, b** Overexpression of circAGAP1 in ACHN and A498 as detected by RT-PCR. Data are presented as the means ± SEM from three independent experiments. **c, d** The viability of A498 and ACHN cells transfected with mock or circAGAP1 as indicated by the CCK-8 assay. **e** EdU analysis and quantification of EdU-positive A498 and ACHN cells transfected with mock or circAGAP1. Cells were subjected to hypoxia for 24 h. **f** Apoptotic rates of A498 and ACHN cells transfected with mock or circAGAP1 as detected by flow cytometry. Cells were subjected to hypoxia for 24 h. **g, h** Transwell assays of A498 and ACHN cells transfected with mock or circAGAP1. The experiments were repeated with at least three biological replicates. **p < 0.01, ***p < 0.001

We further constructed circAGAP1-overexpressing ACHN and A498 cells (Fig. 3a and b, Supplementary Fig. 1b). The results of the CCK-8 and EdU experiments revealed that the viability of ACHN and A498 cells was elevated in the circAGAP1-overexpressing group compared with the mock group (Fig. 3c-e). circAGAP1 overexpression significantly reduced the number of apoptotic cells under hypoxia (Fig. 3f) and dramatically promoted ACHN and A498 cell migration and invasion (Fig. 3g and h). Overall, these data suggest that circAGAP1 is crucial for the viability, invasion and survival of ccRCC cells, which are important for ccRCC progression.

### circAGAP1 directly sponges miR-15-5p in ccRCC cells

**Fig. 4:**
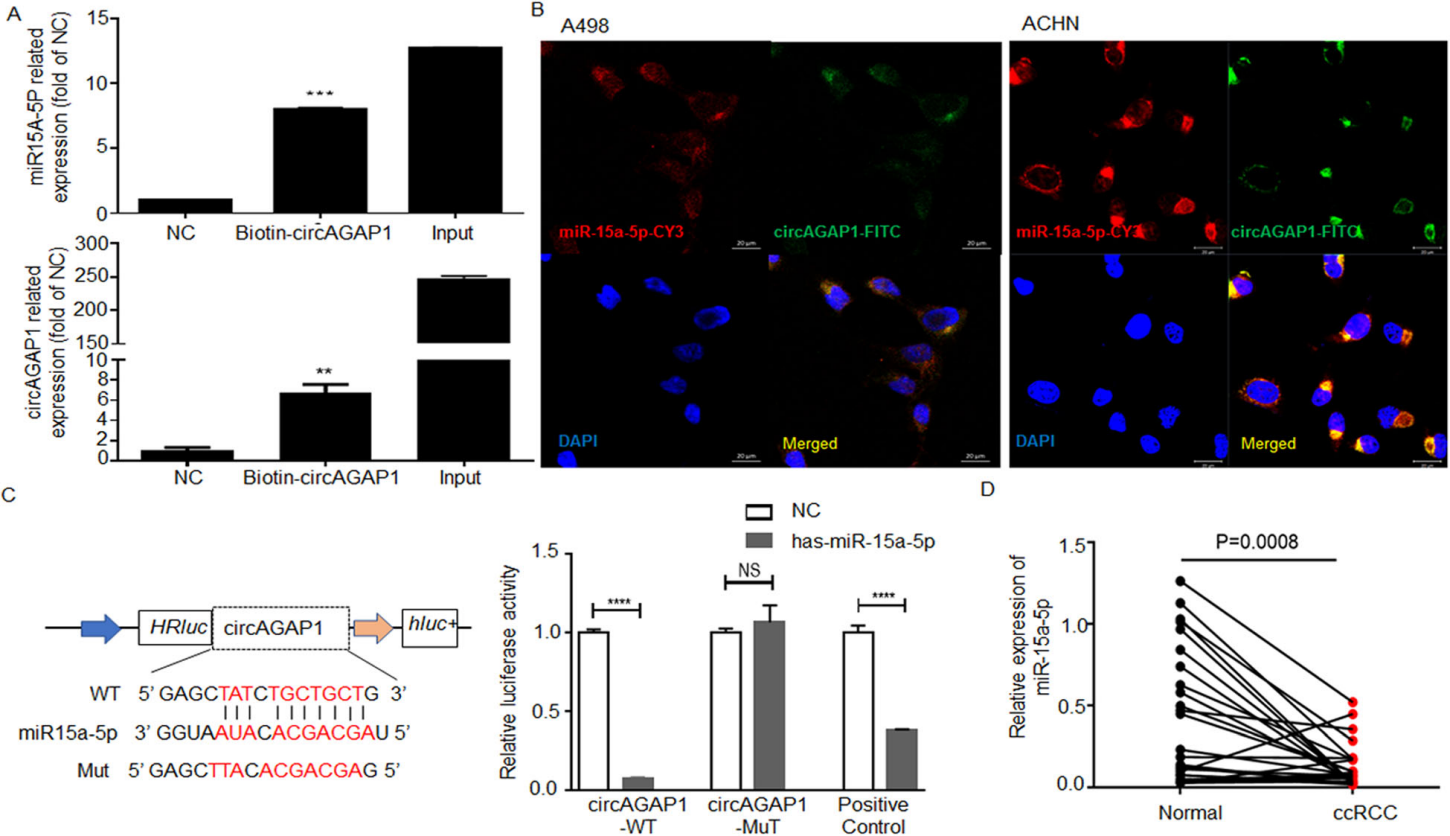
circAGAP1 sponged miR-15a-5p. **a** The interaction between miR-15a-5p and circAGAP1 was assessed by biotin-coupled probe pull-down assay. Data are presented as the means ± SEM from three independent experiments. **b** Detection of colocalization of miR-15a-5p and circAGAP1 in A498 and ACHN cells by RNA-FISH assay. Nuclei were stained with DAPI (magnification, × 400). Red, miR-15a-5p; Green, circAGAP1; Blue, DAPI. **c** Wild-type (WT) and associated mutant (MUT) sequences of the potential miR-15a-5p binding sites in circAGAP1. The effect of the miR-15a-5p mimic on the luciferase activities of WT or MUT circAGAP1 was detected. **d** Expression of miR-15a-5p in ccRCC and adjacent noncancerous tissues was analyzed (*N* = 25, *p* = 0.0008). The experiments were repeated with at least three biological replicates. **p < 0.01, ***p < 0.001, ****p < 0.0001

Studies have revealed that circRNAs sponge miRNAs and abolish the functions of these miRNAs. Thus, we searched miRNAs that could potentially bind circAGAP1 via Arraystar homemade miRNA target prediction software and found that circAGAP1 shares more than one binding site for miR-15a-5p. Thus, to confirm this prediction, a biotin-coupled probe pull-down experiment was applied, and the results revealed that miR-15a-5p and circAGAP1 were present in the pull-down group but not in the control groups (Fig. 4a). Given that circRNAs sponge miRNAs in the cytoplasm, we labeled circAGAP1 (green) and miR-15-5p (red) and observed that they colocalized in the cytoplasm of A498 and ACHN cells (Fig. 4b). Furthermore, luciferase reporter experiment results showed that miR-15-5p mimics significantly inhibited the luciferase activities of the WT reporter but not the MUT reporter (Fig. 4c). Moreover, miR-15a-5p expression was much lower in ccRCC tissues than in paired nontumor tissues (*n* = 34, Fig. 4d).

**Fig. 5:**
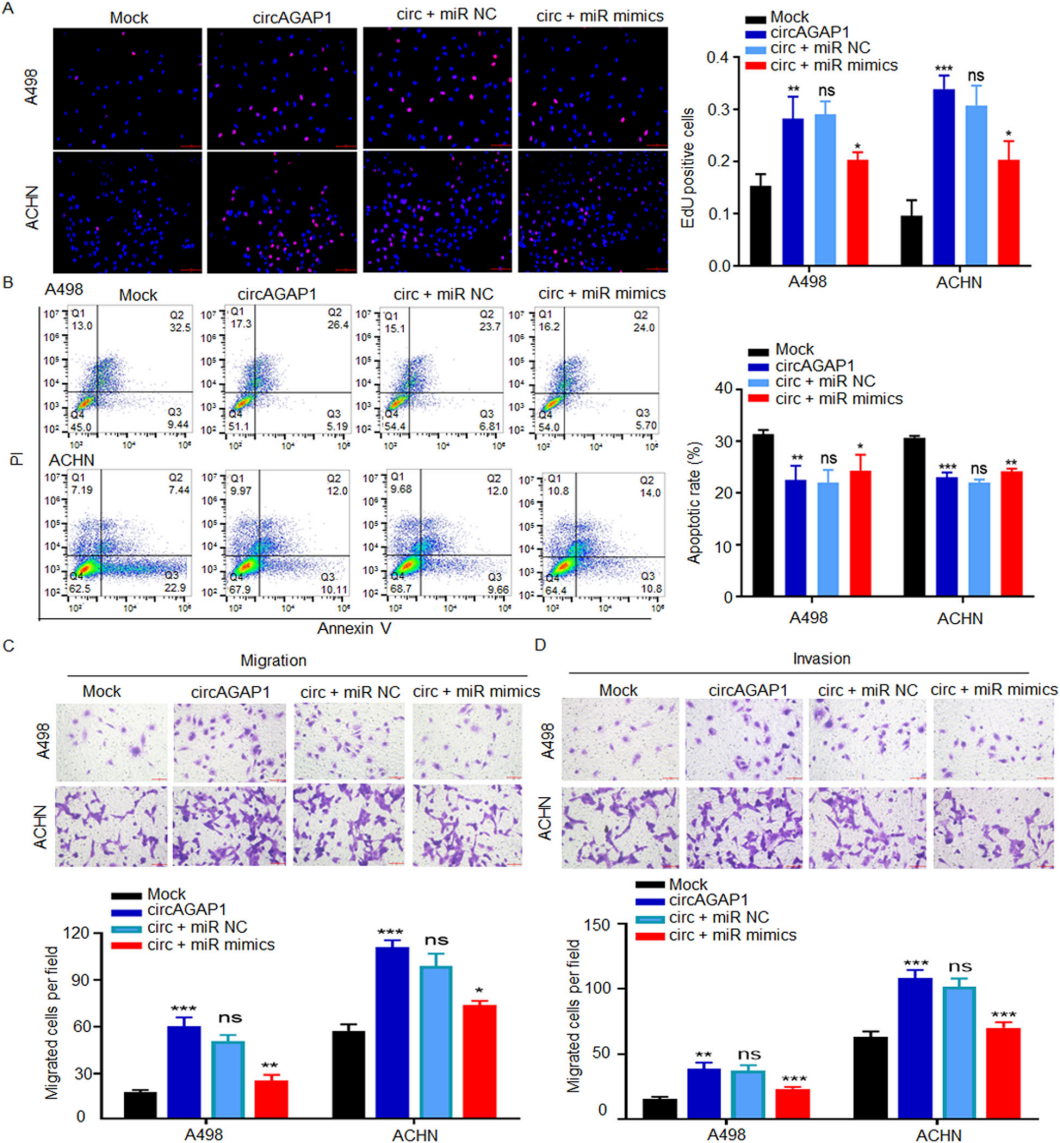
miR-15a-5p partially reversed the circAGAP1-induced proliferation, migration and invasion effects in ccRCC. **a** EdU analysis and quantification of EdU-positive A498 and ACHN cells transfected with mock, circAGAP1, circAGAP1 + miR-NC or circAGAP1 + miR-15a-5p mimics. Cells were subjected to hypoxia for 24 h. **b** Apoptotic rates of A498 and ACHN cells transfected with mock, circAGAP1, circAGAP1 + miR-NC or circAGAP1 + miR-15a-5p mimics as assessed by flow cytometry. Cells were subjected to hypoxia for 24 h. **c, d** Transwell assays of A498 and ACHN cells transfected with mock, circAGAP1, circAGAP1 + miR-NC or circAGAP1 + miR-15a-5p mimics. The experiments were repeated with at least three biological replicates. *p < 0.05, **p < 0.01, ***p < 0.001

To explore whether circAGAP1 regulated malignant behavior in ccRCC by interacting with miR-15a-5p, functional rescue experiments were further devised using miR-15a-5p mimics and conducting EdU assays (Fig. 5a), flow cytometry (Fig. 5b), and Transwell assays (Fig. 5c and d). Cotransfection of miR-15a-5p mimic and circ0058792 partially reversed the increased proliferation, migration and antiapoptotic effects in ACHN and A498 cells (Fig. 5). Altogether, these findings verified the direct interaction between miR-15a-5p and circAGAP1 in ccRCC cells.

### circAGAP1 promotes cellular functions by regulating miR-15-5p/E2F3 in ccRCC cells

**Fig. 6:**
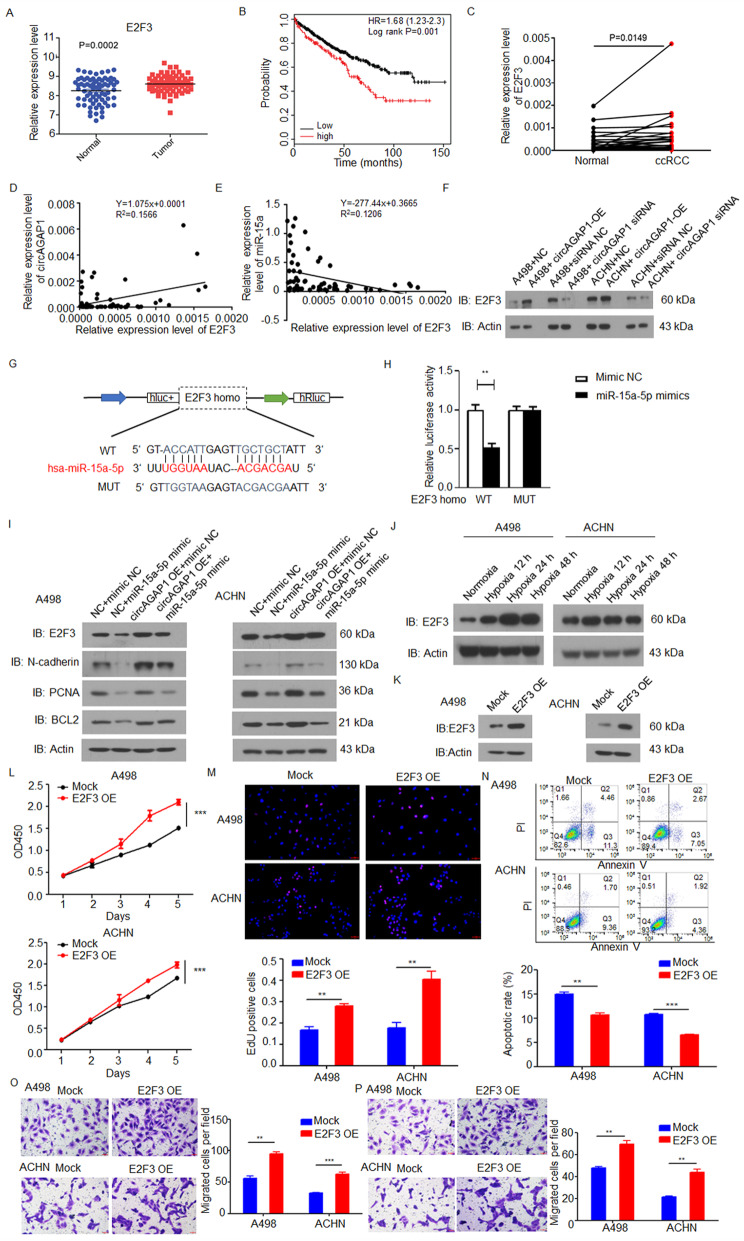
circAGAP1 regulated the miR-15a-5p/E2F3 pathway in ccRCC. a The expression levels of E2F3 in paired ccRCC and adjacent noncancerous tissues were analyzed with samples from the TCGA database (*p* = 0.0002). **b** The relationship between the levels of circAGAP1 and overall survival was analyzed by Kaplan-Meier survival analysis. **C** mRNA levels of E2F3 in ccRCC and adjacent noncancerous tissues detected by qRT-PCR (*n* = 34, *p* = 0.0149). **d, e** Negative associations were found between miR-15a-5p and E2F3 expression in ccRCC, whereas a positive association was found between circAGAP1 and E2F3 expression in ccRCC. **f** The effects of circAGAP1 overexpression (OE) or knockdown (siRNA) on E2F3 protein levels in A498 and ACHN cells detected by Western blotting. **g, h** WT and MUT sequences of the potential binding sites of miR-15a-5p in E2F3. The impact of miR-15a-5p mimics on the luciferase activities of WT-E2F3 and MUT-E2F3 in cells was detected. **i** Influence of circAGAP1 and miR-15a-5p on the protein levels of E2F3, N-cadherin, PCNA, and Bcl-2 in A498 and ACHN cells as detected by WB. **j** Influence of different hypoxia treatments on E2F3 protein levels in A498 and ACHN cells as detected by WB. Cells were subjected to either normoxia or hypoxia for 12 h, 24 h and 48 h. **k** Overexpression of E2F3 in ACHN and A498 cells as detected by WB. **l** The viability of A498 and ACHN cells transfected with mock or E2F3 as detected by CCK-8. **m** EdU analysis and quantification of EdU-positive A498 and ACHN cells transfected with mock or E2F3. Cells were subjected to hypoxia for 24 h. **n** Apoptosis rates of A498 and ACHN cells transfected with mock or E2F3 by flow cytometry. **o, p** Transwell analyses of A498 and ACHN cells transfected with mock or E2F3. The experiments were repeated with at least three biological replicates. **p < 0.01

According to the results of overlapping the prediction of three different databases (miRDIP: http://ophid.utoronto.ca/mirDIP/; TargetScan: http://www.targetscan.org/vert_72/; TarBase: https://www.tarbase.com/), 21 potential miR-15a-5p target genes were found, among which E2F3 was the most likely gene targeted miR-15a-5p in ccRCC (Supplementary Fig. 2). We then examined E2F3 protein expression in ccRCC tissues and found that it was significantly upregulated compared to the levels in adjacent nontumor tissues (Fig. 6a). High E2F3 expression was significantly related to poor prognosis (Fig. 6b). The mRNA expression of E2F3 in ccRCC was also upregulated in ccRCC compared to adjacent nontumor tissues (Fig. 6c). Spearman’s correlation analysis showed that miR-15a-5p was negatively correlated with E2F3 expression, whereas circAGAP1 was positively correlated with E2F3 expression (Fig. 6d and e). The overexpression of circAGAP1 increased the mRNA and protein levels of E2F3, while the suppression of circAGAP1 decreased these levels (Figs. 6f and S3a). The luciferase reporter experiment indicated that compared to the miR-NC, the miR-15-5p mimic dramatically repressed the activity of the E2F3 3′ untranslated region (UTR)-WT sequence (Fig. 6g and h). Furthermore, the miR-15a-5p mimic markedly reduced the expression of E2F3 in ccRCC cells (Figs. 6i and S3b). To verify the regulation of E2F3, we examined the expression of genes downstream of E2F3, including N-cadherin, PCNA and Bcl-2, in A498 and ACHN cells and found that the expression levels of these genes changed consistently with the E2F3 levels in ccRCC (Figs. 6i and S3b). By contrast, we found that ectopic circAGAP1 expression partly rescued the suppressive effect of miR-15a-5p on E2F3 expression in ACHN and A498 cells (Figs. 6i and S3b). In addition, we found that the expression of E2F3 increased under hypoxia stimulation in ccRCC in a time-dependent manner (Figs. 6j and S3c).

We further constructed an E2F3 expression vector and transfected it into A498 and ACHN cells. Both RT-PCR and WB confirmed the success of E2F3 overexpression (Fig. 6k). The results of the CCK-8 and EdU experiments revealed that the viability of ACHN and A498 cells was elevated in the E2F3-overexpressing group compared with the mock group (Fig. 6l and m). E2F3 overexpression significantly reduced the number of apoptotic cells under hypoxia (Fig. 6n) and dramatically promoted ACHN and A498 cell migration and invasion (Fig. 6o and p). Taken together, these data suggest that circAGAP1 could regulate E2F3 by functioning as a ceRNA by sponging miR-15a-5p and that E2F3 promotes ccRCC progression.

### circAGAP1 promotes ccRCC progression in vivo by sponging miR-15a

**Fig. 7:**
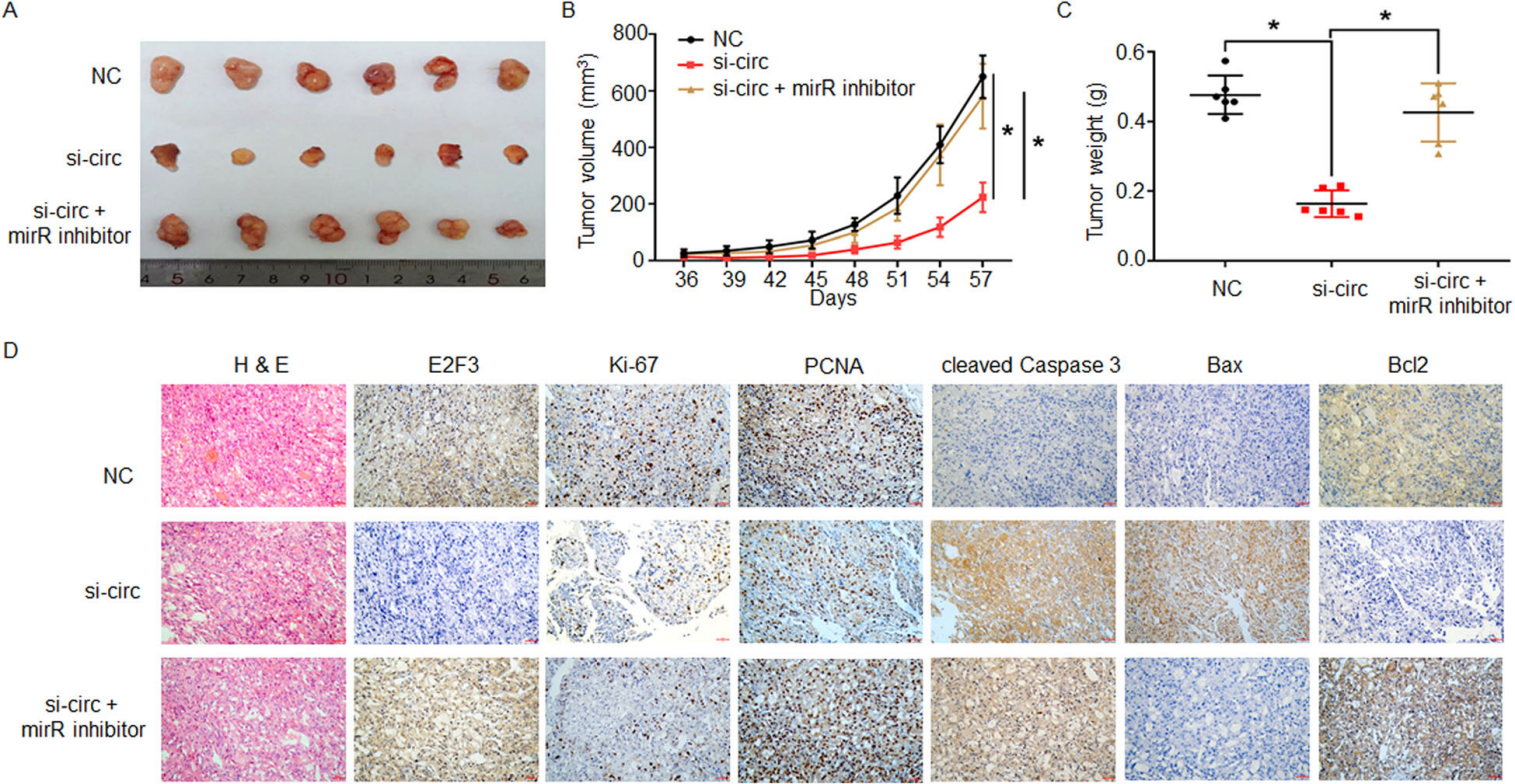
circAGAP1 promoted clear cell renal cell carcinoma progression by targeting miR-15a-5p. **a** Nude mouse models were established via subcutaneous injection of A498 cells. After 4 weeks, the mice were assigned into three groups and intratumorally injected with siRNA NC, circAGAP1 siRNA alone or circAGAP1 siRNA combined with miRNA-15a-5p inhibitors. **b, c** Effects of si-circAGAP1 alone or in combination with miRNA-15a-5p inhibitors on tumor volume and tumor weight. **d** H&E staining displays the morphological landscapes of the inoculated tumors, and an IHC assay was performed to analyze the levels of E2F3, Ki-67, PCNA, cleaved caspase 3, Bax and Bcl-2 in the si-circAGAP1, si-circAGAP1+ miR15a-5p inhibitor and control groups

**Fig. 8:**
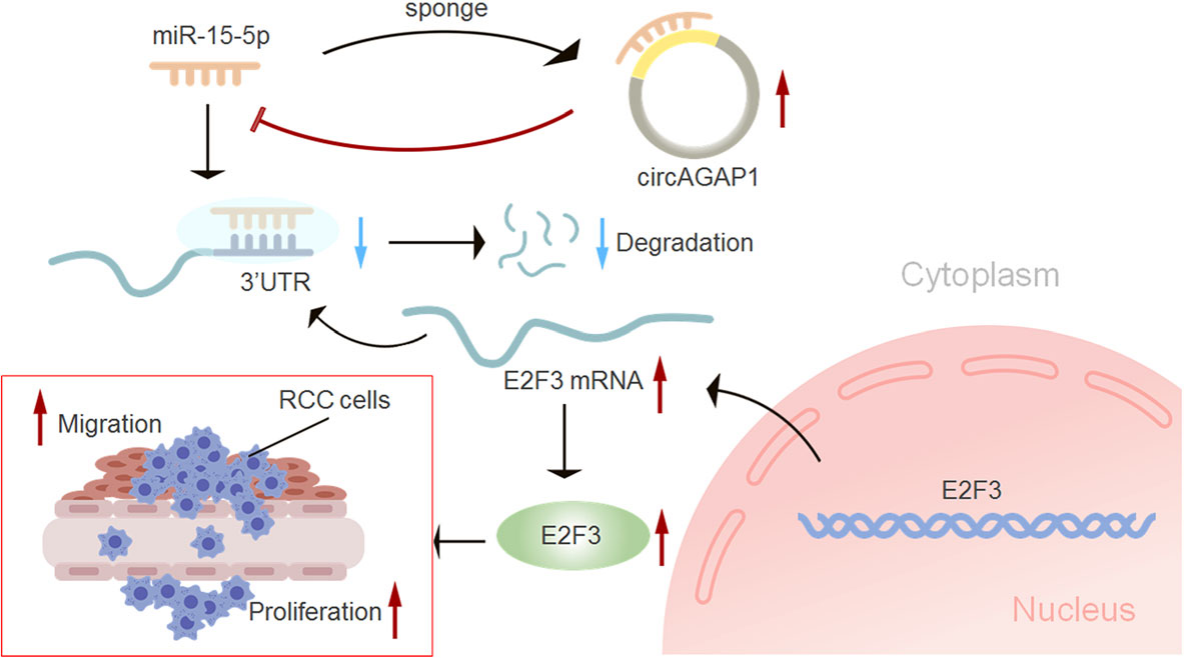
Schematic diagram illustrating the circAGAP1 regulatory pathway in ccRCC cells. circAGAP1 promotes tumor progression by sponging miR-15-5p in clear cell renal cell carcinoma

To identify the relationship between circAGAP1 and the growth and progression of ccRCC in vivo, ACHN cells were subcutaneously injected into nude mice to establish a tumor model, and the mice were treated with circAGAP1 siRNA or siRNA NC. Mice in the circAGAP1 siRNA group had significantly lower mean tumor volumes (*P* < 0.05, Fig. 7a and b) and tumor weights (*P* < 0.05, Fig. 7c) than did mice in the control group. When circAGAP1 siRNA was coinjected with the miR-15a-5p inhibitor, the gains in tumor volume and weight were partly rescued **(**Fig. 7a-c**)**. IHC analysis indicated that the levels of E2F3, Ki-67, PCNA, and Bcl-2 were reduced while those of cleaved caspase 3 and Bax were elevated in the tumors injected with circAGAP1 siRNAs. Intratumoral injection of circAGAP1 siRNA with the miR-15a-5p inhibitor abolished this effect **(**Fig. 7d**)**. Collectively, our results show that the circAGAP1/miR-15a-5p/E2F3 axis promoted the progression of ccRCC (Fig. 8).

## Discussion

In the last few years, several dysregulated circRNAs have been found to be related to tumorigenesis and the progression of tumors such as glioblastoma [6], hepatocellular carcinoma [7, 8], and lung cancer [21]. It is widely recognized that circRNAs are important epigenetic regulators. For example, circ-DONSON is recruited to the NURF complex to promote GC progression by initiating SOX4 expression [22]. circ0091570 expression is downregulated in HCC and functions as a ceRNA by sponging miR-1307 to regulate ISM1 expression [8]. circACAP2 promotes tumorigenesis in breast cancer by modulating the miR-29a/b-3p/COL5A1 axis [23]. CircTP63 promotes squamous cell lung carcinoma progression by regulating FOXM1 [21]. In this study, we revealed that circAGAP1 (circ0058792) was frequently upregulated in ccRCC and highly correlated with worse clinicopathological characteristics. Moreover, silencing circAGAP1 inhibited ccRCC proliferation, migration and invasion, whereas circAGAP1 overexpression further verified the critical functions of circAGAP1 in ccRCC. We experimentally confirmed that circAGAP1 could act as a ceRNA by interacting with miR-15a-5p and consequently blocking it from inhibiting its target gene E2F3. Altogether, this is the first report generally describing the expression, functions, mechanisms and clinical implications of circAGAP1 in ccRCC.

Previous studies have shown that miR-15a-5p participates in the progression of many kinds of tumors in different ways [24]. For example, miR-15a-5p regulates the proliferation and progression of chronic lymphocytic leukemia by suppressing the expression of DLEU2, a host gene of microRNA [25, 26]. miR-15a also regulates the tumorigenesis of multiple myeloma partially by modulating angiogenesis via inhibition of VEGF-A [27]. Largely, miRNAs can bind to the 3′ or 5′-UTR of genes [28–30]. In this study, miR-15a-5p was shown to bind to the 3′-UTR of E2F3, and ectopic expression of miR-15a-5p could partially reverse the malignant behavior of ccRCC by interacting with circAGAP1. This is preliminary evidence that the circAGAP1/miR-15a-5p/E2F3 pathway can regulate the malignant behavior of ccRCC.

According to ceRNA dogma, circRNAs could function as ceRNAs to regulate the activity of target genes [5, 31]. To elucidate the role of circAGAP1 in ccRCC, we analyzed the underlying mechanisms by which circAGAP1 could promote E2F3 expression in ccRCC cells. Consistent with the bioinformatics analysis, dual-luciferase reporter assays confirmed that miR-15a-5p binds to the 3′-UTR of E2F3. Additionally, we found that E2F3 plays an essential role in cell cycle progression, proliferation and development [32]. Our study showed that circAGAP1 inhibition suppressed ccRCC tumor progression through E2F3, further demonstrating the regulatory ability of the circAGAP1/E2F3 axis in ccRCC.

## Conclusions

In summary, our study indicates that upregulated circAGAP1 expression is related to pathological factors associated with poor prognosis in ccRCC. We preliminarily established that circAGAP1 promotes the progression of ccRCC via the circAGAP1/miR-15a-5p/E2F3 pathway. Our study suggests that circAGAP1 is a novel target for RCC treatment.

## Supplementary Information

**Additional file 1: Figure S1.** AGAP1 mRNA levels were not changed in cells transfected with si-circAGAP1. A RT-PCR analysis of AGAP1 expression in ACHN and A498 cells transfected with si-circAGAP1 or si-NC. B RT-PCR analysis of AGAP1 expression in ACHN and A498 cells transfected with mock or circAGAP1. Data are presented as the means ± SEM from three independent experiments.

**Additional file 2: Figure S2.** E2F3 was one of the targets of miR-15a-5p. A Potential miR-15a-5p target genes were predicted by TargetScan, mirDIP, and TarBase. B miR-15a-5p was most positively correlated with E2F3 and most negatively correlated with CSDE1 by bioinformatic analysis.

**Additional file 3: Figure S3.** circAGAP1 regulated E2F3 levels. A Effects of circAGAP1 overexpression or knockdown on E2F3 mRNA levels in A498 and ACHN cells were detected by RT-PCR. B Effects of circAGAP1 and miR-15a-5p on the mRNA levels of E2F3, N-cadherin, PCNA, and Bcl-2 in A498 and ACHN cells were detected by RT-PCR. C Effects of different hypoxia treatments on E2F3 mRNA expression in A498 and ACHN cells were detected by RT-PCR. Data are presented as the means ± SEM from three independent experiments.

**Additional file 4: Supplemental Table 1.** Clinicopathological variables of enrolled ccRCC patients. **Supplemental Table 2.** Association of circAGAP1 expression with clinicopathological variables in ccRCC.

## Data Availability

The datasets supporting the conclusions of this article are included within the article and its additional files.

## Abbreviations

ccRCC: Clear cell renal cell carcinoma
circRNA: Circular RNA
FISH: Fluorescence in situ hybridization
IHC: Immunohistochemistry
PCR: Polymer chain reaction
ceRNA: Competitive endogenous RNA
UTR: Untranslated region
E2F3: E2F transcription factor 3
PCNA: Proliferating cell nuclear antigen

## References

[1] Bray F, Ferlay J, Soerjomataram I, Siegel RL, Torre LA, Jemal A (2018). Global cancerstatistics 2018: GLOBOCAN estimates of incidence and mortality worldwide for 36cancers in 185 countries. CA A Cancer J Clin.

[2] Powles T, Staehler M, Ljungberg B (2016). European Association of Urology guidelines for clear cell renal cancers that are resistant to vascular endothelial growth factor receptor-targeted therapy. Eur Urol.

[3] Kotecha RR, Motzer RJ, Voss MH (2019). Towards individualized therapy for metastatic renal cell carcinoma. Nat Rev Clin Oncol.

[4] Mahmoudi E, Kiltschewskij D, Fitzsimmons C (2019). Depolarization-associated CircRNA regulate neural gene expression and in some cases may function as templates for translation. Cells..

[5] Li R, Jiang J, Shi H, Qian H (2020). CircRNA: a rising star in gastric cancer. Cell Mol Life Sci.

[6] Wang R, Zhang S, Chen X, Li N, Li J, Jia R, Pan Y, Liang H (2018). CircNT5E Acts as a Sponge of miR-422a to Promote Glioblastoma Tumorigenesis. Cancer Res.

[7] Yu J, Xu QG, Wang ZG (2018). Circular RNA cSMARCA5 inhibits growth and metastasis in hepatocellular carcinoma. J Hepatol.

[8] Han D, Li J, Wang H, Su X, Hou J, Gu Y, Qian C, Lin Y, Liu X, Huang M, Li N, Zhou W, Yu Y, Cao X (2017). Circular RNA circMTO1 acts as the sponge of microRNA-9 to suppress hepatocellular carcinoma progression. Hepatology..

[9] Huang E, Liu R, Chu Y (2015). miRNA-15a/16: as tumor suppressors and more. Future Oncol.

[10] Leng J, Song Q, Zhao Y (2018). miR-15a represses cancer cell migration and invasion under conditions of hypoxia by targeting and downregulating Bcl-2 expression in human osteosarcoma cells.[J]. Int J Oncol.

[11] Sirotkin Alexander V, Kisová G, Brenaut P (2014). Involvement of microRNA Mir15a in control of human ovarian granulosa cell proliferation, apoptosis, steroidogenesis, and response to FSH. Microrna.

[12] Lin K, Farahani M, Yang Y (2014). Loss of MIR15A and MIR16–1 at 13q14 is associated with increased TP53 mRNA, de-repression of BCL2 and adverse outcome in chronic lymphocytic leukaemia. Br J Haematol.

[13] Chava S, Reynolds CP, Pathania AS (2020). miR-15a-5p, miR-15b-5p, and miR-16-5p inhibit tumor progression by directly targeting MYCN in neuroblastoma. Mol Oncol.

[14] Guo S, Fesler A, Huang W, Wang Y (2020). Functional significance and therapeutic potential of miR-15a mimic in pancreatic ductal adenocarcinoma. Mol Ther Nucleic Acids.

[15] Woods K, Thomson JM, Hammond SM (2007). Direct regulation of an oncogenic micro-RNA cluster by E2F transcription factors. J Biol Chem.

[16] Huang E, Ishida S, Pittman J (2003). Gene expression phenotypic models that predict the activity of oncogenic pathways. Nat Genet.

[17] Shan Z-N, Tian R, Zhang M (2016). miR128–1 inhibits the growth of glioblastoma multiforme and glioma stem-like cells via targeting BMI1 and E2F3. Oncotarget.

[18] Yu R, Cai L, Chi Y (2018). miR-377 targets CUL4A and regulates metastatic capability in ovarian cancer. Int J Mol Med.

[19] Han R, Zhao J, Lu L (2020). MicroRNA-34a expression affects breast cancer invasion in vitro and patient survival via downregulation of E2F1 and E2F3 expression. Oncol Rep.

[20] Song P, Ye L-F, Zhang C (2016). Long non-coding RNA XIST exerts oncogenic functions in human nasopharyngeal carcinoma by targeting miR-34a-5p. Gene.

[21] Cheng Z, Yu C, Cui S, Wang H, Jin H, Wang C, Li B, Qin M, Yang C, He J, Zuo Q, Wang S, Liu J, Ye W, Lv Y, Zhao F, Yao M, Jiang L, Qin W. circTP63 functions as a ceRNA to promote lung squamous cell carcinoma progression by upregulating FOXM1. Nat Commun. 2019;10(1):3200. 10.1038/s41467-019-11162-4. 10.1038/s41467-019-11162-4 PMC6642174 31324812

[22] Ding L, Zhao Y, Dang S, Wang Y, Li X, Yu X, Li Z, Wei J, Liu M, Li G (2019). Circular RNA circ-DONSON facilitates gastric cancer growth and invasion via NURF complex dependent activation of transcription factor SOX4. Mol Cancer.

[23] Zhao B, Song X, Guan H (2020). CircACAP2 promotes breast cancer proliferation and metastasis by targeting miR-29a/b-3p-COL5A1 axis. Life Sci.

[24] Aqeilan RI, Calin GA, Croce CM (2010). miR-15a and miR-16-1 in cancer: discovery, function and future perspectives. Cell Death Differ.

[25] Klein U, Lia M, Crespo M (2010). The DLEU2/miR-15a/16-1 cluster controls B cell proliferation and its deletion leads to chronic lymphocytic leukemia. Cancer Cell.

[26] Lerner M, Harada M, Lovén J (2009). DLEU2, frequently deleted in malignancy, functions as a critical host gene of the cell cycle inhibitory microRNAs miR-15a and miR-16-1. Exp. Cell Res.

[27] Sun C-Y, She X-M, Qin Y (2013). miR-15a and miR-16 affect the angiogenesis of multiple myeloma by targeting VEGF.[J]. Carcinogenesis.

[28] Pillai RS, Bhattacharyya SN, Filipowicz W (2007). Repression of protein synthesis by miRNAs: how many mechanisms. Trends Cell Biol.

[29] Vishnoi A, Rani S (2017). MiRNA biogenesis and regulation of diseases: an overview. Methods Mol Biol.

[30] Lu TX, Rothenberg ME (2018). MicroRNA. J Allergy Clin Immunol.

[31] Liu Y, Yang Y, Wang Z (2020). Insights into the regulatory role of circRNA in angiogenesis and clinical implications. Atherosclerosis..

[32] Wu L, Timmers C, Maiti B (2001). The E2F1-3 transcription factors are essential for cellular proliferation. Nature.

