# Supplementary figures and images for: CircAGAP1 promotes tumor progression by sponging miR-15-5p in clear cell renal cell carcinoma
Source: J Exp Clin Cancer Res. 2021 Feb 22;40:76. doi: 10.1186/s13046-021-01864-3 (PMC7901094; doi:10.1186/s13046-021-01864-3)

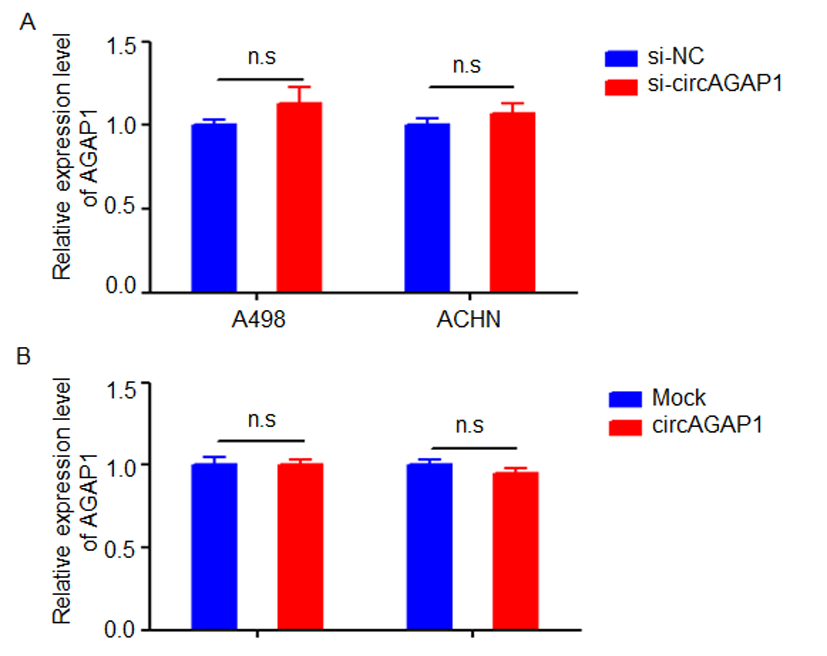

Supplement: Supplementary file 1 — Additional file 1: Figure S1. AGAP1 mRNA levels were not changed in cells transfected with si-circAGAP1. A RT-PCR analysis of AGAP1 expression in ACHN and A498 cells transfected with si-circAGAP1 or si-NC. B RT-PCR analysis of AGAP1 expression in ACHN and A498 cells transfected with mock or circAGAP1. Data are presented as the means ± SEM from three independent experiments. [file 13046_2021_1864_MOESM1_ESM.tif]

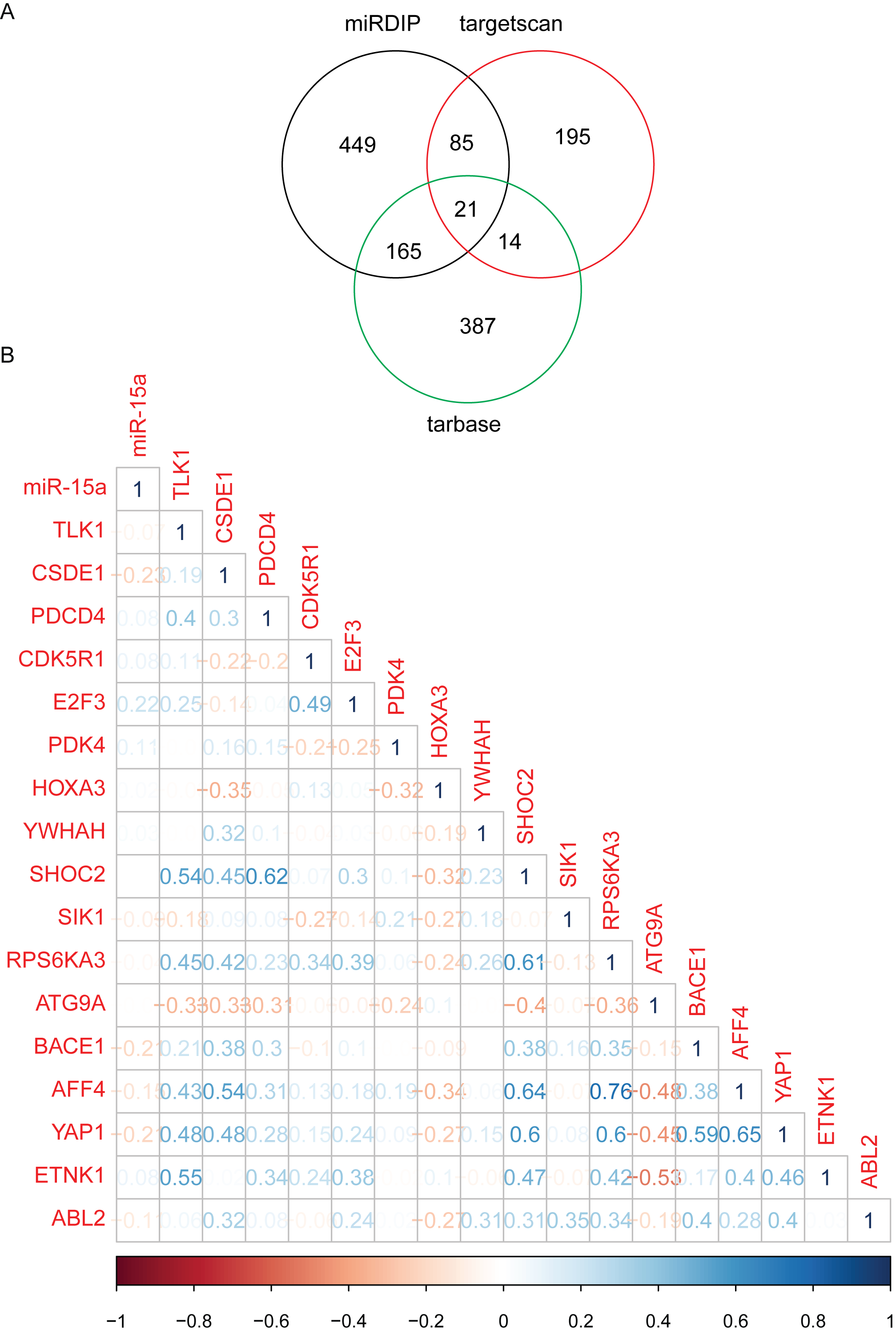

Supplement: Supplementary file 2 — Additional file 2: Figure S2. E2F3 was one of the targets of miR-15a-5p. A Potential miR-15a-5p target genes were predicted by TargetScan, mirDIP, and TarBase. B miR-15a-5p was most positively correlated with E2F3 and most negatively correlated with CSDE1 by bioinformatic analysis. [file 13046_2021_1864_MOESM2_ESM.tif]

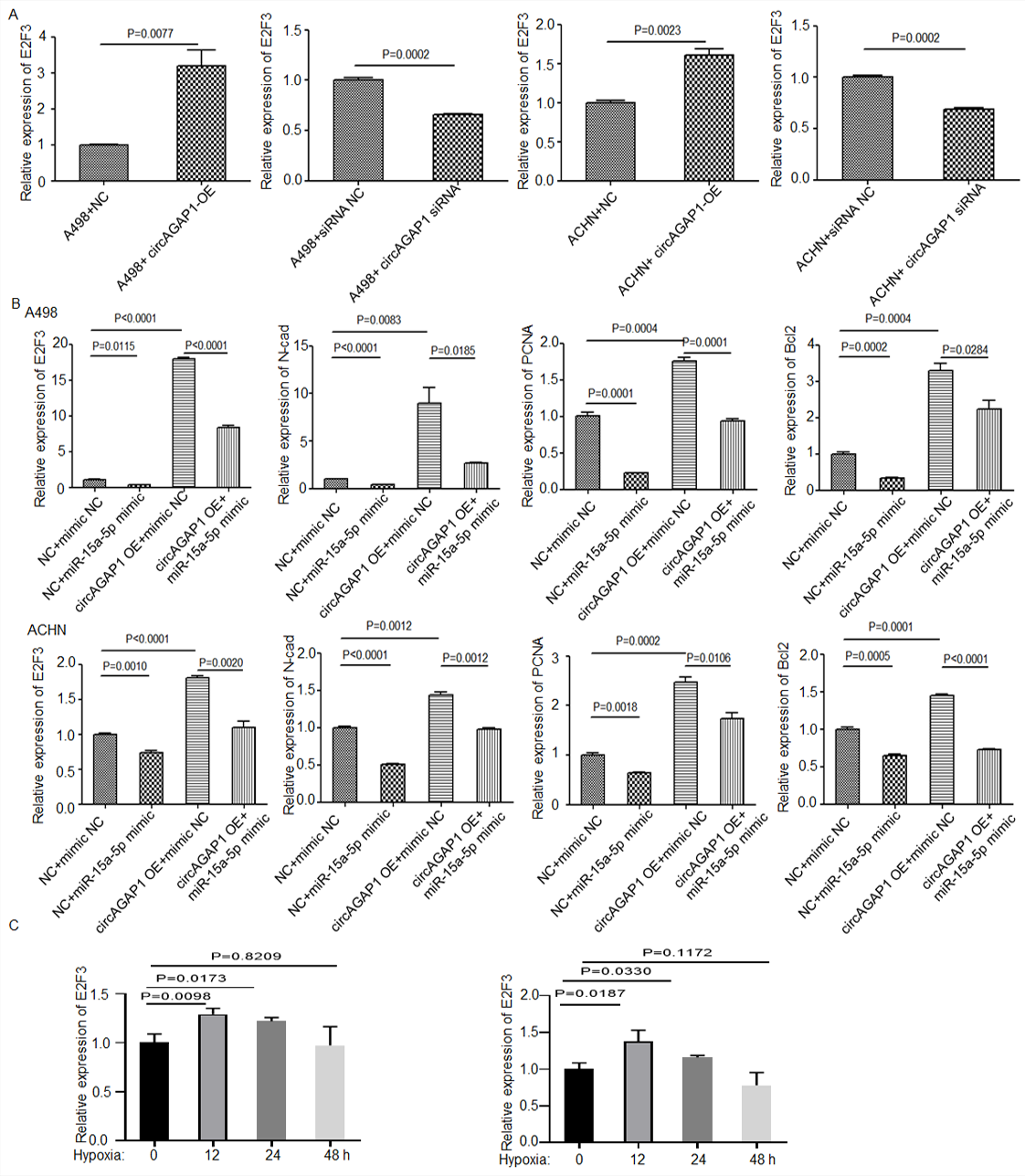

Supplement: Supplementary file 3 — Additional file 3: Figure S3. circAGAP1 regulated E2F3 levels. A Effects of circAGAP1 overexpression or knockdown on E2F3 mRNA levels in A498 and ACHN cells were detected by RT-PCR. B Effects of circAGAP1 and miR-15a-5p on the mRNA levels of E2F3, N-cadherin, PCNA, and Bcl-2 in A498 and ACHN cells were detected by RT-PCR. C Effects of different hypoxia treatments on E2F3 mRNA expression in A498 and ACHN cells were detected by RT-PCR. Data are presented as the means ± SEM from three independent experiments. [file 13046_2021_1864_MOESM3_ESM.tif]
