# Supplementary material for: CircAGAP1 promotes tumor progression by sponging miR-15-5p in clear cell renal cell carcinoma
Source: J Exp Clin Cancer Res. 2021 Feb 22;40:76. doi: 10.1186/s13046-021-01864-3 (PMC7901094; doi:10.1186/s13046-021-01864-3)
Supplement: Supplementary file 4 — Additional file 4: Supplemental Table 1. Clinicopathological variables of enrolled ccRCC patients. Supplemental Table 2. Association of circAGAP1 expression with clinicopathological variables in ccRCC. [file 13046_2021_1864_MOESM4_ESM.docx]

**Supplemental tables**

Supplemental table 1. Clinicopathological variables of enrolled ccRCC patients.

| NO. | gender | age | Symptom/Physical signs | Left/right kidney | size | nuclear grade | TNM (clinical)stage | Lymphatic Metastasis |
| --- | --- | --- | --- | --- | --- | --- | --- | --- |
| 1 | male | 73 | None | left | 4.2cm×4.3cm | G2 | T_1b_N_0_M_0_(Ⅰ) | NO |
| 2 | female | 74 | Hematuria/abdominal palpable mass | right | 12cm×8.6cm | G4 | T_3a_N_1_M_0_(Ⅲ) | YES |
| 3 | male | 67 | None | right | 7.5cm×5.2cm | G3 | T_2_N_1_M_0_(Ⅲ) | YES |
| 4 | male | 61 | None | left | 3.6cm×2.9cm | G1-2 | T_1a_N_0_M_0_(Ⅰ) | NO |
| 6 | male | 62 | None | right | 4.9cm×4.5cm | G3 | T_1a_N_0_M_0_(Ⅰ) | NO |
| 7 | male | 73 | None | right | 2.7cm×2.4cm | G2 | T_1a_N_0_M_0_(Ⅰ) | NO |
| 8 | male | 51 | Fever | left | 10cm×7.2cm | G3 | T_3a_N_1_M_0_(Ⅲ) | YES |
| 9 | male | 70 | None | left | 1.6cm×1.6cm | G2 | T_1a_N_0_M_0_(Ⅰ) | NO |
| 10 | male | 73 | None | right | 4.0cm×3.5cm | G2 | T_1a_N_0_M_0_(Ⅰ) | NO |
| 11 | male | 63 | None | left | 5.6cm×5.0cm | G3 | T_1b_N_0_M_0_(Ⅰ) | NO |
| 12 | male | 70 | Hematuria&Fever | right | 9.5cm×9.0cm | G3-4 | T_2_N_0_M_1_(Ⅳ) | NO |
| 13 | male | 78 | Hematuria/abdominal palpable mass | left | 4.5cm×4.6cm | G3 | T_1b_N_0_M_0_(Ⅰ) | NO |
| 14 | male | 47 | None | right | 3.6cm×3.1cm | G2 | T_1a_N_0_M_0_(Ⅰ) | NO |
| 15 | male | 73 | None | right | 4 cm×3.6cm | G2 | T_1a_N_1_M_0_(Ⅲ) | YES |
| 16 | female | 52 | None | right | 3cm×3cm | G2 | T_1a_N_0_M_0_(Ⅰ) | NO |
| 17 | male | 57 | Macroscopic hematuria | left | 9.5cm×8.6cm | G2 | T_2_N_0_M_1_(Ⅳ) | NO |
| 18 | female | 49 | None | left | 2.6cm×2.5cm | G1 | T_1a_N_0_M_0_(Ⅰ) | NO |
| 20 | female | 46 | None | right | 3.7cm×3.1cm | G1 | T_1a_N_1_M_0_(Ⅲ) | YES |
| 21 | male | 52 | None | left | 2.8cm×2.2cm | G1-2 | T_1a_N_0_M_0_(Ⅰ) | NO |
| 22 | male | 63 | Back pain | left | 6.7cm×5.1cm | G2 | T_1b_N_1_M_0_(Ⅲ) | YES |
| 23 | male | 63 | Hematuria/abdominal palpable mass | right | 9.6cm×7.5cm | G3 | T_2_N_1_M_1_(Ⅳ) | YES |
| 25 | male | 73 | None | right | 5.9cm×5.7cm | G3 | T_1a_N_0_M_0_(Ⅰ) | NO |
| 28 | female | 47 | None | right | 5.1cm×5.7cm | G3 | T_1a_N_0_M_0_(Ⅰ) | NO |
| 29 | female | 74 | Macroscopic hematuria | left | 11cm×10cm | G2 | T_2_N_1_M_0_(Ⅲ) | YES |
| 30 | female | 52 | None | right | 3.0cm×2.7cm | G1 | T_1a_N_0_M_0_(Ⅰ) | NO |
| 31 | female | 72 | None | right | 8 cm×7.5cm | G3 | T_2_N_1_M_0_(Ⅲ) | YES |
| 32 | male | 58 | None | right | 6.5cm×5.9cm | G3 | T_1b_N_0_M_0_(Ⅰ) | NO |
| 34 | male | 59 | None | left | 6.3cm×5.1cm | G1-2 | T_1a_N_0_M_0_(Ⅰ) | NO |
| 35 | female | 80 | None | right | 2.5cm×2.0cm | G1-2 | T_1a_N_0_M_0_(Ⅰ) | YES |
| 36 | male | 42 | None | right | 4.7cm×4.5cm | G3-4 | T_1a_N_0_M_0_(Ⅰ) | NO |
| 37 | female | 60 | None | right | 1.5cm×1.5cm | G1 | T_1a_N_0_M_0_(Ⅰ) | NO |
| 38 | female | 38 | None | left | 6.5cm×5.5cm | G1-2 | T_1b_N_0_M_1_(Ⅳ) | NO |
| 39 | female | 65 | None | right | 1.8cm×1.6cm | G1-2 | T_1a_N_0_M_0_(Ⅰ) | NO |
| 57 | male | 56 | Back pain | left | 8.6cm×8.3cm | G3-4 | T_2a_N_0_M_0_(II) | NO |

Supplemental table 2. Association of circAGAP1 expression with clinicopathological variables in ccRCC.

| Characteristics | No. Patients（%） | mean±SD | | *T* value | | *p* value | |  |
| --- | --- | --- | --- | --- | --- | --- | --- | --- |
| Sex |  |  | |  | |  | |  |
| Female | 11(33.33) | 0.0008±0.013 | | 0.281 | | 0.780 | |  |
| Male | 22(66.67) | 0.0010±0.002 | |  |  |  |  |  |
| Age (years old) |  |  | |  | |  | |  |
| ＜60 | 13(39.39) | 0.0006±0.0009 | | | 0.654 | | 0.518 | |
| ≥60 | 20(60.61) | 0.0012±0.0026 | | |  |  |  | |
| Tumor Size |  |  |  |  | |  | |  |
| ≥5 | 14 (42.42) | 0.0020±0.0030 | | 2.633 | | 0.013 | |  |
| <5 | 19 (57.58) | 0.0002±0.0003 | |  |  |  |  |  |
| nuclear grade |  |  |  |  | |  | |  |
| G1-2 | 20 (60.60) | 0.0004±0.0006 | | 2.039 | |  | |  |
| G3-4 | 13（39.40） | 0.0019±0.0032 | |  |  | 0.050 | |  |
| clinical Stage |  |  |  |  | |  | |  |
| Ⅰ+Ⅱ | 23（66.67） | 0.0002±0.0005 | | 3.128 | | 0.004 | |  |
| Ⅲ+Ⅳ | 10（33.33） | 0.0024± 0.0033 | |  |  |  |  |  |
| Lymphatic Metastasis |  |  |  |  | |  | |  |
| NO | 23（69.70） | 0.0008±0.0024 | | 0.578 | | 0.568 | |  |
| YES | 10（30.30） | 0.0013±0.0014 | |  |  |  |  |  |
